# A novel inflammatory-nutritional index: NPAR-correlates with the severity of type 2 diabetic foot ulcers

**DOI:** 10.3389/fnut.2026.1855891

**Published:** 2026-06-18

**Authors:** Kai Lin, Xiong Lei, Hongzhe Wang, Danhong Zhang, Ru Wang, Xingxing Zhang, Cai Lin

**Affiliations:** 1 Department of Burn and Wound Healing Center, The First Affiliated Hospital of Wenzhou Medical University, Wenzhou, China; 2 Department of Emergency, The First Affiliated Hospital of Wenzhou Medical University, Wenzhou, China; 3 Department of Endocrinology, The First Affiliated Hospital of Wenzhou Medical University, Wenzhou, China; 4 Wenzhou Medical University, The First School of Medicine, School of Information and Engineering, Wenzhou, China

**Keywords:** albumin, inflammatory-nutritional index, neutrophil, neutrophil-percentage-to-albumin ratio, type 2 diabetic foot ulcer severity, Wagner classification

## Abstract

**Background:**

The neutrophil-percentage-to-albumin ratio (NPAR), an inflammatory-nutritional biomarker, has prognostic value in various diseases, but its relationship with DFU severity is unclear. This study investigates the association between NPAR and DFU severity.

**Methods:**

This study was a retrospective cross-sectional study that analyzed clinical data from 1,221 consecutive hospitalized patients with type 2 diabetes mellitus (T2DM) foot lesions admitted to the Burn and Trauma Center of the First Affiliated Hospital of Wenzhou Medical University between January 2020 and January 2025. The severity of diabetic foot ulcers was graded using the Wagner classification system. Ordinal logistic regression, restricted cubic spline analysis, and subgroup analyses were applied to assess the association of NPAR with DFU severity.

**Results:**

Participants were stratified into NPAR quartiles: Q1 (≤17.89), Q2 (17.89–21.36), Q3 (21.36–25.81) and Q4 (≥25.82). Higher NPAR values paralleled greater Wagner grades, longer hospital stays, higher costs and more frequent surgical procedures. In the unadjusted model, each one-unit rise in NPAR conferred an odds ratio (OR) of 1.197 (95% CI, 1.170–1.225) for advanced DFU severity. After full adjustment for 17 covariates, the OR remained 1.174 (95% CI, 1.143–1.205, *p* < 0.001). Across quartiles, a pronounced dose–response relationship was observed: subjects in Q4 exhibited an 11.3-fold higher risk of severe DFU relative to Q1 (OR = 11.344; 95% CI, 7.621–16.886). RCS analysis revealed a non-linear threshold effect; the risk of worsening DFU escalated sharply once NPAR exceeded 21.4. Trend tests indicated a 2.214-fold incremental risk per quartile (P-for-trend < 0.001). Subgroup analyses confirmed the stability of these associations irrespective of sex, age, smoking, alcohol consumption or hypertension status.

**Conclusion:**

NPAR is independently and robustly associated with DFU severity in a non-linear dose-dependent manner. As an inexpensive and readily obtainable inflammatory-nutritional composite index, NPAR may help assess the severity of DFU at presentation and assist in clinical decision-making, thereby supplementing clinical decision-making.

## 1 Introduction

Diabetic foot ulcer (DFU) is the most frequent and devastating chronic complication of type 2 diabetes mellitus (T2DM), affecting approximately 6.3% of the global diabetic population; its five-year mortality is 2.5-fold higher than that of diabetic patients without foot lesions (1, 2). DFU arises from the interplay of neuropathy, peripheral arterial disease and infection, and may ultimately lead to amputation, impaired quality of life and premature death (3, 4). Although glycaemic control, advanced wound care and antimicrobial strategies have improved, clinical outcomes remain suboptimal. There is an urgent need for more sensitive and readily available biomarkers to identify high-risk patients early and guide individualized interventions (5, 6).

Chronic low-grade inflammation and malnutrition are central to the pathogenesis of diabetes and its complications. Patients with diabetic foot ulcers (DFU) often exhibit a persistent state of low-grade inflammation, characterized by elevated levels of inflammatory cytokines such as C-reactive protein (CRP), interleukin-6 (IL-6) in the circulation. This inflammatory response subsequently inhibits angiogenesis and delays wound healing (7–9). Meanwhile, malnutrition is also highly prevalent among DFU patients. The common hypoalbuminemia in these patients is not only associated with delayed wound healing and increased infection risk, but also closely linked to elevated risks of amputation and mortality (10–12). There exists a bidirectional regulatory relationship between inflammation and nutritional status: inflammation can induce albumin catabolism and inhibit its synthesis, while malnutrition may exacerbate immune dysfunction, creating a vicious cycle (13–16).

Neutrophils constitute the first line of innate immunity and play a pivotal role in the local and systemic inflammatory response observed in DFU (17). Peripheral neutrophil counts are increased in affected patients; these cells release neutrophil extracellular traps and reactive oxygen species that amplify tissue injury and retard re-epithelialisation (9). Serum albumin is a reliable marker of nutritional reserve that also exerts antioxidant, endothelial-protective and immunomodulatory functions. Low albumin concentrations correlate strongly with DFU severity, infection burden and adverse prognosis (10, 13). A composite index integrating neutrophil burden and albumin availability may therefore offer superior discriminative power.

The neutrophil-percentage-to-albumin ratio (NPAR) is a novel inflammatory-nutritional index that has recently demonstrated robust prognostic performance in critical illness, cardiovascular disease and malignancy (18–20). NPAR can simultaneously reflect the intensity of inflammatory activation and the adequacy of nutrient reserves. Compared to single inflammatory or nutritional indicators, it offers more stable detection, lower costs, and universal accessibility, giving it unique advantages in resource-limited environments.

Although the prognostic value of NPAR in various diseases has been preliminarily validated, its relationship with diabetic foot ulcers (DFU) remains inadequately studied systematically. A recent cross-sectional study revealed for the first time a significant positive correlation between NPAR and DFU prevalence (21). However, research specifically addressing the severity of diabetic foot ulcers remains limited.

The assessment of diabetic foot ulcer severity is crucial for guiding treatment plans and predicting prognosis in patients with diabetic foot. Currently, the clinical evaluation of diabetic foot ulcer severity is a relatively complex process, requiring comprehensive consideration of multiple factors such as wound morphology, neuropathic, vascular, and skeletal lesions of the foot, as well as infection status, and heavily relies on the experience of clinicians. Therefore, there is an urgent need for novel objective indicators to assist clinicians in evaluating or predicting the severity of diabetic foot ulcers.

To our knowledge, no study has examined whether NPAR correlates with DFU severity. We hypothesised that higher NPAR values would parallel more advanced ulceration. Accordingly, the present cross-sectional retrospective analysis was undertaken to delineate the association between NPAR and DFU severity in a large, consecutive cohort of hospitalised patients with T2DM.

## 2 Methods

### 2.1 Study population

**Figure 1:**
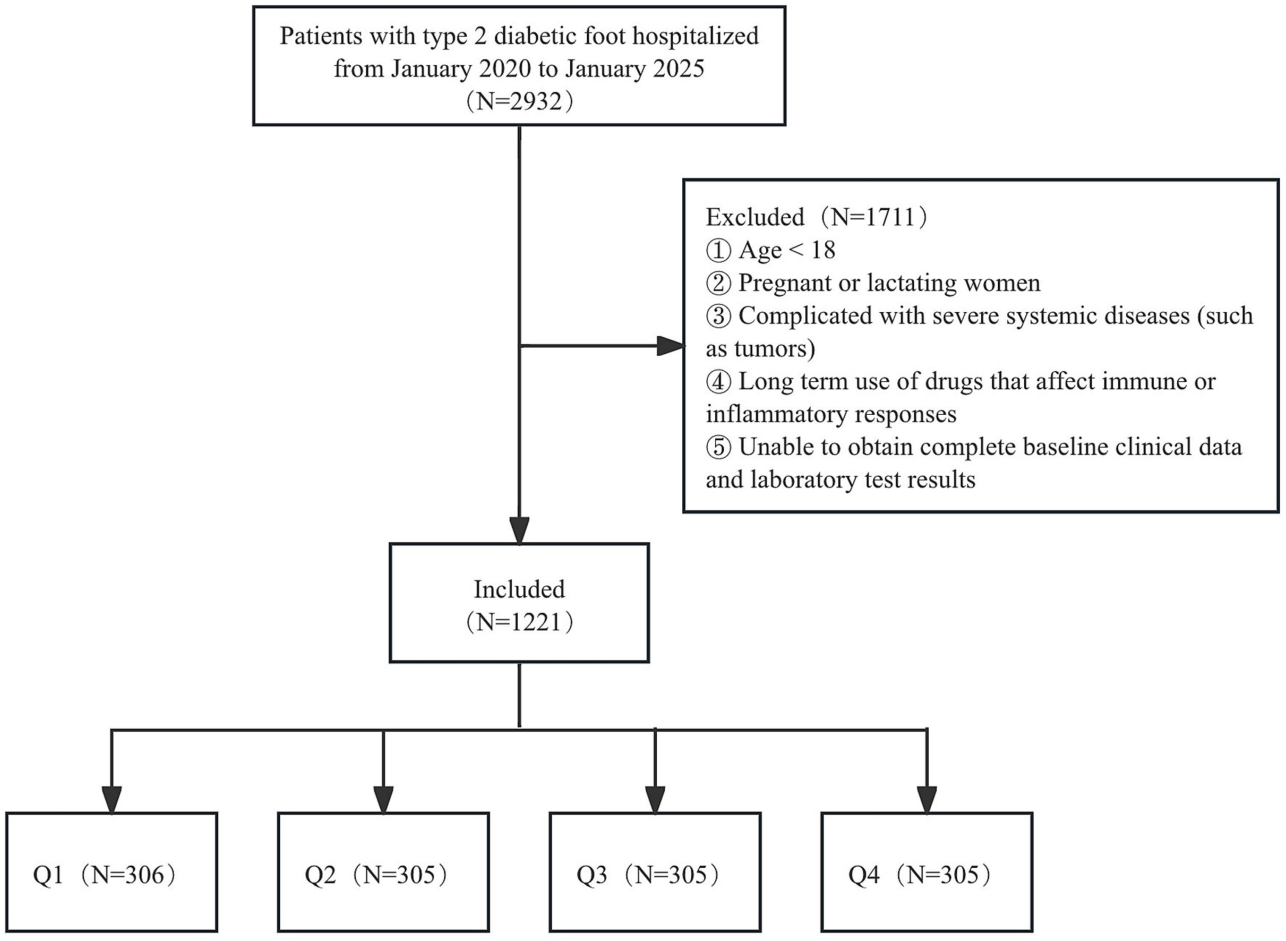
Flowchart of patient selection.

To guarantee both rigour and generalisability, eligibility was determined through pre-defined inclusion and exclusion criteria. Consecutive patients admitted to the Burn & Wound Centre of the First Affiliated Hospital of Wenzhou Medical University between January 2020 and January 2025 were screened. Inclusion criteria were: (i) in-patient management for a foot lesion related to type 2 diabetes mellitus, (ii) availability of complete clinical and laboratory records. Individuals were excluded if they were aged <18 years, pregnant or lactating, suffering from severe heart failure (NYHA class III-IV), end-stage liver disease (Child-Pugh class C), autoimmune diseases, and other conditions, recent glucocorticoid or immunosuppressive therapy, or missing key data (e.g., serum albumin and neutrophil percentage). Application of these criteria identified 1,221 eligible participants from an initial pool of 2,932 (Figure 1).

### 2.2 Exposure definition

The NPAR was defined as an exposure factor in this study. The NPAR was calculated as neutrophil percentage (%) divided by serum albumin concentration (g/dL).

### 2.3 Outcome definition

The outcome of this study was DFU severity. DFU severity was graded using the validated Wagner classification. For the primary analysis, we pre-specified a three-category grouping based on clinical relevance: mild (Wagner grades 0–2, superficial ulcers without deep tissue involvement), moderate (grade 3, deep ulcer with abscess, osteomyelitis, or joint involvement), and severe (grades 4–5, localized or extensive gangrene).

### 2.4 Variable selections

In this study, covariates potentially associated with the outcome of interest (DFU severity) were incorporated as comprehensively as possible to control for potential confounding. Covariate selection was based on *a priori* clinical knowledge and previously published literature. Demographic factors included sex and age. Lifestyle factors comprised smoking history and alcohol consumption, both of which have been demonstrated to be associated with endocrine and metabolic disorders (22, 23). In addition, a common chronic condition, namely a history of hypertension, was included as a covariate. Regarding laboratory parameters, the following variables were included: hemoglobin (Hb), fasting glucose (Glu), total cholesterol (TC), triglycerides (TG), high-density lipoprotein cholesterol (HDL-C), low-density lipoprotein cholesterol (LDL-C), international normalized ratio (INR), total bilirubin (TBIL), aspartate aminotransferase (AST), alanine aminotransferase (ALT), estimated glomerular filtration rate (eGFR), and serum creatinine (Crea). These indicators reflect the patient’s anemia status (Hb), glycemic control (Glu), lipid metabolism (TC, TG, HDL-C, LDL-C), coagulation function (INR), liver function (TBIL, AST, ALT), and kidney function (eGFR, Crea). Previous studies have shown that the above laboratory parameters are closely associated with systemic inflammatory status, nutritional reserve, and the severity of diabetic complications (including diabetic foot) in patients with diabetes (24–26). Therefore, they were entered into the multivariate model to control for potential confounding effects.

### 2.5 Data collection and definitions

Clinical data were retrieved retrospectively from the electronic medical record system of the First Affiliated Hospital of Wenzhou Medical University. Demographic characteristics (sex, age), lifestyle factors (smoking and alcohol consumption), medical history (hypertension), hospitalisation cost, length of stay and number of surgical procedures were extracted. Laboratory variables were obtained from venous blood samples drawn after an overnight fast (>8 h). Measured parameters comprised haemoglobin (Hb), fasting glucose (Glu), total cholesterol (TC), triglycerides (TG), high-density lipoprotein cholesterol (HDL-C), international normalised ratio (INR), total bilirubin (TBIL), aspartate aminotransferase (AST), alanine aminotransferase (ALT), serum creatinine (Crea) and estimated glomerular filtration rate (eGFR). We also extracted additional clinical information from the electronic medical record system, including patients’ chief complaints at admission, medical visit history, physical examination findings, and intraoperative observations. These data provided descriptions of infection status, wound size, wound morphology, osteomyelitis, and other relevant features, which were used to assist the researchers in improving the accuracy of diabetic foot ulcer severity assessment.

### 2.6 Statistical analysis

Baseline characteristics were summarised across NPAR quartiles. Normality was examined with the Kolmogorov–Smirnov test. Normally distributed continuous variables are presented as mean ± SD and compared with one-way ANOVA; non-normally distributed variables are expressed as median (inter-quartile range) and compared with the Kruskal–Wallis test. Categorical data are given as number (percentage) and evaluated by *χ*^2^ or Mantel–Haenszel *χ*^2^ test as appropriate. The association between NPAR (either as a continuous variable or by quartiles) and Wagner grade was examined with ordinal logistic regression, yielding odds ratios (OR) and 95% confidence intervals (CI). Finally, two groups of multivariate regression models were constructed, and the Brant test was used to examine the proportional odds assumption of the ordered logistic regression models. For continuous NPAR and for quartiles, model 1 was unadjusted; model 2 added sex and age; model 3 further adjusted smoking, alcohol consumption and hypertension; model 4 additionally included all 17 laboratory covariates. A restricted cubic spline (RCS) with four knots was fitted to visualise the dose–response relationship, and trend tests across quartiles were performed. Subgroup analyses stratified by age, sex, smoking, alcohol use and hypertension were undertaken to assess consistency of the main effect. All analyses were conducted with R version 4.3.2; two-sided *p* < 0.05 denoted statistical significance.

## 3 Results

### 3.1 Baseline characteristics

Participants were divided into quartiles according to NPAR: Q1 (≤17.89), Q2 (17.89–21.36), Q3 (21.36–25.81) and Q4 (≥25.82). Table 1 summarises the clinical profile of each group. Higher NPAR values were associated with a greater proportion of high Wagner grades, increased hospitalisation costs, longer length of stay and a higher number of surgical procedures during the index admission (all *p* < 0.001). Across quartiles, significant differences were also observed for age, haemoglobin, fasting glucose, total cholesterol, triglycerides, HDL-cholesterol, LDL-cholesterol, international normalised ratio, aspartate aminotransferase, alanine aminotransferase, estimated glomerular filtration rate and serum creatinine (all *p* < 0.001). In contrast, sex distribution, smoking status, alcohol consumption, prevalence of hypertension and total bilirubin did not differ among groups (all *p* > 0.05).

**Table 1:** Clinical characteristics based on NPAR quartiles.

Variable	Q1 (*N* = 306)	Q2 (*N* = 305)	Q3 (*N* = 305)	Q4 (*N* = 305)	*p*-value
Age (years)	67.00 [58.00, 75.00]	71.00 [63.00, 76.00]	69.00 [61.00, 75.00]	66.00 [58.00, 74.00]	<0.001
Sex (%)					0.445
Female	106 (34.6)	90 (29.5)	89 (29.2)	95 (31.1)	
Male	200 (65.4)	215 (70.5)	216 (70.8)	210 (68.9)	
Smoking status (%)					0.065
No	226 (73.9)	209 (68.5)	210 (68.9)	234 (76.7)	
Yes	80 (26.1)	96 (31.5)	95 (31.1)	71 (23.3)	
Drinking status (%)					0.938
No	222 (72.5)	218 (71.5)	218 (71.5)	224 (73.4)	
Yes	84 (27.5)	87 (28.5)	87 (28.5)	81 (26.6)	
Hypertension (%)					0.756
No	154 (50.3)	143 (46.9)	146 (47.9)	141 (46.2)	
Yes	152 (49.7)	162 (53.1)	159 (52.1)	164 (53.8)	
Wagner grading (%)					<0.001
0	2 (0.7)	2 (0.7)	0 (0.0)	0 (0.0)	
1	24 (7.8)	2 (0.7)	0 (0.0)	0 (0.0)	
2	119 (38.9)	81 (26.6)	43 (14.1)	9 (3.0)	
3	114 (37.3)	130 (42.6)	111 (36.4)	76 (24.9)	
4	47 (15.4)	90 (29.5)	151 (49.5)	218 (71.5)	
5	0 (0.0)	0 (0.0)	0 (0.0)	2 (0.7)	
Hospitalization expenses	19685.44 [13750.5, 29447.3]	24936.73 [17165.9, 34767.4]	27454.13 [18049.1, 38362.5]	39498.41 [24775.6, 52825.8]	<0.001
Hospital stays (days)	9.00 [6.00, 13.00]	10.00 [7.00, 14.00]	10.00 [7.00, 16.00]	14.00 [9.00, 20.00]	<0.001
Number of surgeries	1.00 [1.00, 2.00]	1.00 [1.00, 2.00]	1.00 [1.00, 2.00]	2.00 [1.00, 2.00]	<0.001
Hb (g/L)	120.50 [109.00, 135.00]	116.00 [102.00, 125.00]	106.00 [94.00, 119.00]	96.00 [84.00, 111.00]	<0.001
Glu (mmol/L)	10.90 [7.50, 14.40]	10.70 [7.50, 15.10]	12.10 [8.10, 16.40]	14.30 [10.10, 19.90]	<0.001
TC (mmol/L)	4.44 [3.59, 5.29]	4.08 [3.39, 4.96]	3.86 [3.14, 4.60]	3.47 [2.81, 4.47]	<0.001
TG (mmol/L)	1.66 [1.10, 2.50]	1.39 [1.04, 2.03]	1.24 [0.98, 1.69]	1.25 [0.96, 1.76]	<0.001
HDL-C (mmol/L)	0.99 [0.85, 1.18]	0.93 [0.79, 1.12]	0.86 [0.70, 1.03]	0.72 [0.57, 0.89]	<0.001
LDL-C (mmol/L)	2.67 [2.08, 3.27]	2.50 [1.95, 3.13]	2.39 [1.84, 2.94]	2.22 [1.69, 2.75]	<0.001
INR	1.00 [0.96, 1.04]	1.04 [0.98, 1.10]	1.07 [1.02, 1.13]	1.14 [1.08, 1.22]	<0.001
TBIL (μmol/L)	8.00 [6.00, 11.00]	8.00 [7.00, 10.00]	8.00 [6.00, 11.00]	8.00 [6.00, 11.00]	0.601
AST (U/L)	20.00 [16.00, 26.00]	18.00 [14.00, 24.00]	17.00 [14.00, 22.00]	18.00 [13.00, 27.00]	<0.001
ALT (U/L)	18.00 [13.00, 26.75]	15.00 [11.00, 24.00]	15.00 [10.00, 21.00]	15.00 [10.00, 25.00]	<0.001
eGFR	86.30 [62.70, 104.68]	73.30 [40.60, 97.70]	72.30 [26.00, 96.20]	74.50 [27.90, 97.80]	<0.001
Crea (μmol/L)	73.00 [58.00, 98.00]	83.00 [65.00, 133.00]	90.00 [66.00, 191.00]	87.00 [63.00, 198.00]	<0.001

HB, hemoglobin, Glu, glucose, TC, total cholesterol, TG, triglyceride, HDL-C, high-density lipoprotein cholesterol, LDL-C, low-density lipoprotein cholesterol, INR, International standardized ratio, TBIL, serum total bilirubin, AST, aspartate aminotransferase, ALT, alanine aminotransferase, eGFR, estimated glomerular filtration rate, Crea, Creatinine.

### 3.2 Association between NPAR (continuous and quartiles) and DFU severity: ordinal logistic regression

A cross all models, NPAR was positively and significantly associated with Wagner grade (Table 2). In the unadjusted model (model 1), each one-unit increment in NPAR conferred a 19.7% increase in the odds of a higher Wagner grade (OR = 1.197; 95% CI, 1.170–1.225, *p* < 0.001). Adjustment for age and sex (model 2) did not materially alter the estimate (OR = 1.198; 95% CI, 1.170–1.225, *p* < 0.001). The association remained essentially unchanged after additional adjustment for lifestyle and clinical risk factors (model 3: OR = 1.198; 95% CI, 1.171–1.226, *p* < 0.001) and was only modestly attenuated in the fully adjusted model incorporating 17 laboratory covariates (model 4: OR = 1.174; 95% CI, 1.143–1.205, p < 0.001).

**Table 2:** The relationship between NPAR and the severity of DFUs.

Exposure	Severity of DFUs, OR (95% CI)							
	Model 1	P value	Model 2	P value	Model 3	P value	Model 4	*p*-value
NPAR (Continue)	1.197 (1.170, 1.225)	<0.001	1.198 (1.170, 1.225)	<0.001	1.198 (1.171,1.226)	<0.001	1.174 (1.143, 1.205)	<0.001
NPAR quartiles								
Q1 (8.96–17.89)	Reference		Reference		Reference		Reference	
Q2 (17.89–21.36)	2.369 (1.751, 3.204)	<0.001	2.300 (1.694, 3.122)	<0.001	2.274 (1.675, 3.088)	<0.001	2.001 (1.461, 2.741)	<0.001
Q3 (21.36–25.81)	5.656 (4.127, 7.752)	<0.001	5.611 (4.088, 7.702)	<0.001	5.570 (4.057, 7.648)	<0.001	4.220 (3.001, 5.934)	<0.001
Q4 (25.82–49.26)	15.667 (11.092, 22.127)	<0.001	15.842 (11.207, 22.394)	<0.001	16.102 (11.378, 22.787)	<0.001	11.344 (7.621, 16.886)	<0.001

NPAR, Neutrophil-percentage-to-albumin ratio; OR, odds ratio; HB, hemoglobin; Glu, glucose; TC, total cholesterol; TG, triglyceride; HDL-C, high-density lipoprotein cholesterol; LDL-C, low-density lipoprotein cholesterol; INR, International standardized ratio; TBIL, serum total bilirubin; AST, aspartate aminotransferase; ALT, Alanine Aminotransferase; eGFR, estimated glomerular filtration rate; Crea, Creatinine.

Model 1 was unadjusted.

Model 2 was adjusted for age, sex.

Model 3 was adjusted for age, sex, smoke, drink, hypertension.

Model 4 was adjusted for age, sex, smoke, drink, hypertension, eGFR, LDLC, TC, Hb, Glu, TG, HDLC, INR, TBIL, AST, ALT, Crea.

When NPAR was analysed in quartiles, a strong dose–response relationship was evident across all models (Table 2), and the graded association remained robust after comprehensive multivariable adjustment (model 4). Participants in Q4 had an 11.3-fold higher odds of increased DFU severity compared with those in Q1 (OR = 11.344; 95% CI, 7.621–16.886, *p* < 0.001), whereas patients in Q2 and Q3 exhibited intermediate risk elevations (Q2: OR = 2.001; 95% CI, 1.461–2.741; Q3: OR 4.220, 95% CI 3.001–5.934; both *p* < 0.001).

The proportional odds assumption for the ordered logistic regression model was checked using the Brant test, which showed no significant violation (*p* > 0.05).

### 3.3 Dose–response relationship between NPAR and DFU severity

Restricted cubic spline analysis, fully adjusted for age, sex, lifestyle factors and laboratory variables, revealed a pronounced non-linear dose–response relationship between continuous NPAR and DFU severity (Supplementary Figure S1). Knots positioned at the 10th, 50th and 90th percentiles (15.8, 21.4 and 31.4, respectively) identified critical transition points. Once NPAR exceeded the median value of 21.4, the risk of progression to severe DFU rose steeply.

### 3.4 Trend test across NPAR quartiles

A significant linear trend was observed: each stepwise increase in quartile was associated with a 2.214-fold rise in the odds of more severe DFU (95% CI, 1.952–2.511, P-for-trend < 0.001). The observed ORs (red dots) aligned closely with the fitted linear trend (green line) depicted in Supplementary Figure S2.

### 3.5 Subgroup analysis

**Figure 2:**
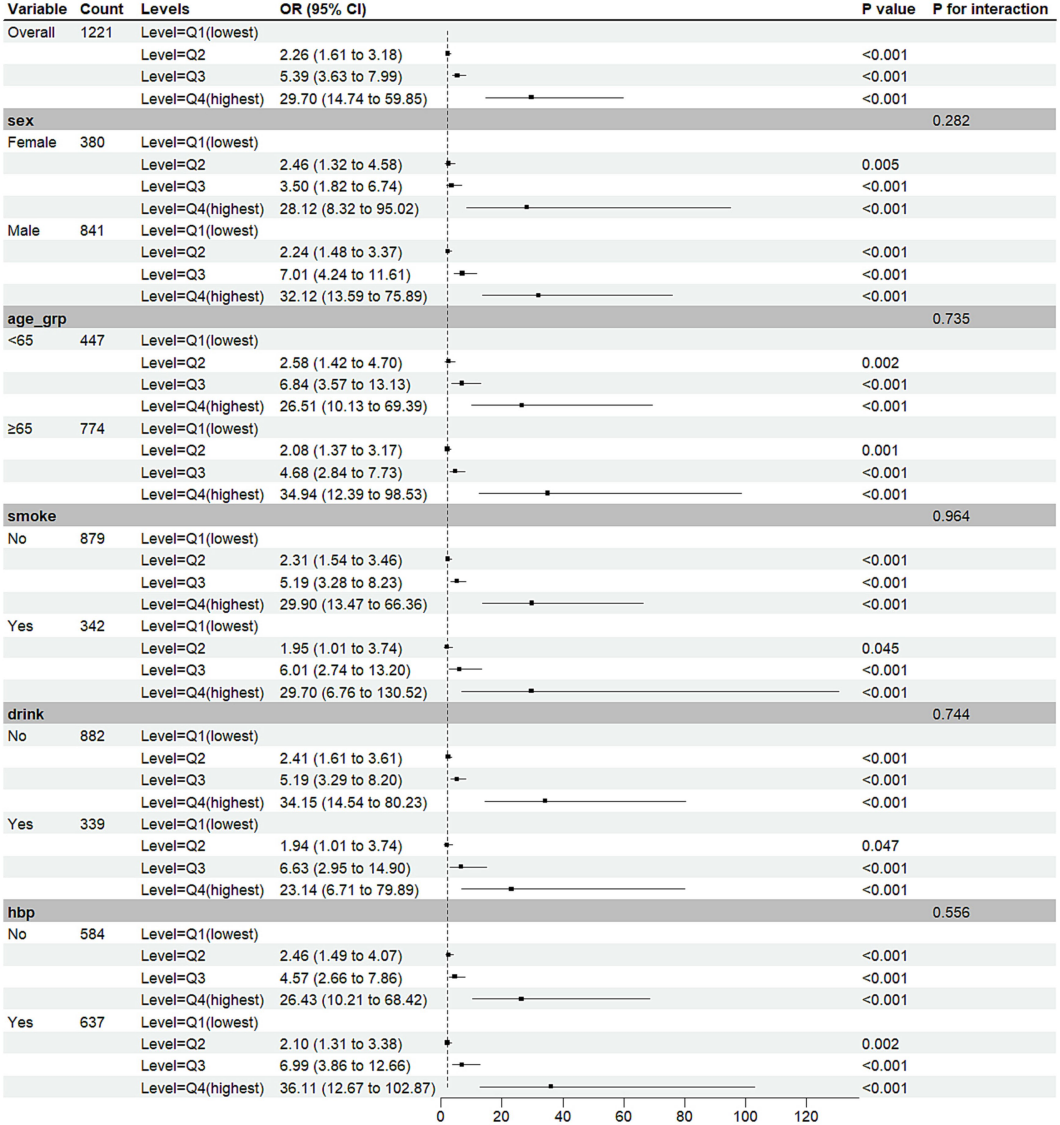
Forest plots in different subgroups.

To examine whether the association between NPAR and DFU severity was modified by other risk factors, we performed subgroup analyses stratified by sex, age (< 65 vs. ≥ 65 years), smoking status, alcohol consumption and history of hypertension (Figure 2). A significant dose–response relationship across NPAR quartiles was observed in every stratum. The risk of advanced DFU rose consistently with increasing quartiles, indicating that the strong link between NPAR and ulcer severity remains stable irrespective of demographic or clinical characteristics.

## 4 Discussion

### 4.1 Main results

This large, cross-sectional study of 1,221 hospitalised patients with T2DM foot disease provides the first systematic evidence that the novel inflammatory-nutritional index NPAR is significantly and robustly associated with DFU severity. Three principal findings emerge. First, after full adjustment for 17 potential confounders, each one-unit increment in NPAR was linked to a 17.4% increase in the odds of severe DFU (Wagner grades 4–5; OR 1.174). Second, quartile analysis revealed a pronounced dose–response gradient: participants in the highest quartile (Q4: ≥25.82) exhibited an 11.3-fold higher risk of severe DFU compared with those in the lowest quartile (Q1: ≤17.89), and trend tests confirmed a linear upward trajectory. Third, restricted cubic spline analysis identified a critical non-linear threshold, once NPAR exceeded the median value of 21.4, the risk of advanced DFU escalated exponentially. These observations remained consistent across subgroups defined by sex, age, lifestyle factors and comorbidities, underscoring the potential utility of NPAR as an objective severity-stratification tool for diabetic foot disease.

### 4.2 Comparison and interpretations

The findings both echo and extend previous reports on NPAR in diabetes-related complications. He XJ, Tan JT, Wang SY et al. demonstrated positive associations between elevated NPAR and diabetic kidney disease, peripheral arterial disease and retinopathy, with satisfactory discriminative performance (27–30). Subsequent studies by Li, Ji, Jing et al. further linked higher NPAR to increased all-cause mortality in diabetic cohorts (31–34). Most pertinent to the present work, Chen H using NHANES data, observed that the highest NPAR tertile conferred a 73% higher odds of DFU prevalence (OR = 1.73); however, their analysis was confined to a binary “ulcer present/absent” outcome and did not adjust for key laboratory confounders such as eGFR or liver function (21). By adopting the finer-grained Wagner ordinal scale, we not only corroborate the cross-sectional association of NPAR with DFU severity but also reveal a pronounced gradient across the severity continuum. The magnitude of risk amplification underscores the superior discriminative power of NPAR when applied to clinical severity rather than simple presence of ulceration. Of note, the Q1–Q3 quartiles exhibited an almost perfect dose–linearity (Q2: OR = 2.001; Q3: OR = 4.220), whereas Q4 showed a sharp inflection to 11.344. RCS analysis confirmed this J-shaped pattern, revealing a threshold effect at approximately 21.4, beyond which the inflammation-nutrition imbalance may enter a decompensated stage, leading to accelerated wound deterioration. These data provide an evidence-based cut-point for clinical risk alerting, which can be externally validated in an independent cohort in the future.

Compared with other inflammatory–nutritional composite indices, NPAR offers a theoretical advantage by simultaneously capturing the “driver” of immune activation (neutrophil percentage) and the “buffer” of systemic reserve (serum albumin). In the pathophysiology of DFU, persistent hyperglycaemia triggers aberrant neutrophil activation, with release of neutrophil extracellular traps (NETs) and reactive oxygen species (ROS) that sustain a pro-inflammatory milieu, whereas hypoalbuminaemia impairs collagen synthesis and vascular repair (13, 35, 36). Traditional single markers such as C-reactive protein or albumin are valuable but vulnerable to acute infection (CRP fluctuation) or volume distribution (albumin dilution). The ratio structure of NPAR partially neutralises these sources of variability, conferring enhanced stability. The strong correlations observed in our cohort between NPAR and both length of stay and number of surgical procedures indirectly support the association of this composite index with these clinically relevant outcomes.

The robust link between NPAR and DFU severity may be underpinned by several interconnected mechanisms. First, neutrophils are not merely infection markers; they are the first leukocytes to accumulate in diabetic target tissues such as pancreas and insulin-sensitive organs (5, 37, 38). In patients with diabetes mellitus or prediabetes, chronic inflammatory states and insulin resistance caused by obesity or prolonged hyperglycemia can lead to an increase in neutrophile granulocyte, with alterations in both the phenotype and function of these cells (5, 39). The significant correlations of NPAR with eGFR and aminotransferases further imply that systemic inflammatory load may aggravate metabolic dysfunction via a “renal–hepatic axis,” perpetuating a vicious cycle (40, 41). Second, albumin fulfils roles that extend far beyond nutritional assessment. As the most abundant circulating protein (13, 42), it binds and transports drugs and metabolites, maintains oncotic pressure to facilitate re-absorption of wound exudate (43), scavenges ROS released by NETs (44, 45), and exerts chaperone activity that prevents misfolding and aggregation of damaged proteins while promoting clearance of aged fibrinogen and fibrin (46, 47). It is important to note, however, that serum albumin levels in hospitalized DFU patients are not determined solely by nutritional status. Systemic inflammation, illness severity, and reduced hepatic synthetic function due to comorbid conditions all contribute to hypoalbuminemia. Therefore, NPAR should be interpreted as an integrated inflammatory-nutritional index rather than a pure nutrition marker. By integrating these complementary biological processes into a single value, NPAR effectively quantifies the net balance between “inflammatory injurious force” and “tissue reparative capacity”. A ratio exceeding 21.4 likely denotes a potential tipping point at which reparative reserves can no longer counterbalance inflammatory injury, mirroring the clinical transition to chronic, non-healing ulcers. The precise tipping point requires further investigation and validation in more comprehensive clinical cohorts.

Our findings provide a novel, bedside-compatible instrument for rapid risk stratification and identify patients with greater severity. NPAR is calculated from only two routinely available tests making it inexpensive and universally accessible across all levels of care. In patients with Wagner grades 0–2, an NPAR in the Q4 range (≥25.82) should prompt heightened vigilance for progression to severe ulceration; intensified glycaemic control, early nutritional support (protein intake >1.5 g kg^−1^ day^−1^) and proactive debridement are then indicated. Conversely, individuals in the Q1 group may be safely followed at extended intervals, thereby optimising resource allocation. Importantly, the dynamic monitoring potential of NPAR deserves emphasis. Although the present study is cross-sectional, existing evidence indicates that combined nutritional and anti-inflammatory interventions can significantly lower NPAR. Future interventional trials should explore whether NPAR-guided, individualised therapy can improve outcomes and whether serial NPAR measurements can function as a “treatment-response biomarker”.

In the context of infected DFU, NPAR may also inform antimicrobial stewardship. Conventional infection markers such as CRP exhibit limited specificity for diabetic foot osteomyelitis (48), whereas NPAR-by simultaneously incorporating inflammatory and nutritional dimensions-could more accurately identify patients who require prolonged antibiotic courses. The positive correlation observed in our cohort between NPAR and the number of surgical procedures further suggests that NPAR might predict the need for operative intervention. Prospective cohorts are now warranted to validate whether NPAR can serve as an adjunctive tool for amputation-risk stratification.

### 4.3 Strengths and limitations

Principal strengths of this investigation lie in methodological rigour and clinical relevance. The sample size (*n* = 1,221) provided ample statistical power, ensuring robust estimates even within the Wagner 4–5 subgroup. Data quality was safeguarded by extracting 17 covariates-covering demographics, lifestyle and laboratory domains-from the electronic medical record; the fully adjusted model minimised residual confounding. Analytically, the combined use of ordinal logistic regression, restricted cubic splines and trend tests allowed us to characterise the exposure–response relationship from linear, non-linear and dose-gradient perspectives, offering substantially greater informational depth than conventional binary logistic approaches. Most importantly, we propose, for the first time, a “threshold effect” for NPAR in DFU severity assessment, thereby furnishing a clear hypothesis for future mechanistic studies.

Several limitations merit cautious interpretation. First of all, cross-sectional research and design cannot determine the time sequence; At present, it is not clear whether the increase of NPAR is a precursor of more serious DFU, triggered by the latter, or driven by upstream factors such as chronic hyperglycemia. And NPAR was calculated based on a single blood sample obtained at the time of admission. We did not monitor the continuous changes of NPAR during hospitalization or after treatment. Therefore, we cannot evaluate whether the decline of NPAR is related to clinical improvement, or whether the rising NPAR indicates bad results such as nonunion or amputation. Prospective cohort study is needed to measure the baseline NPAR level, record the longitudinal changes of NPAR and track the new or progressive DFU to clarify the causal relationship. Secondly, although the Wagner classification is currently a widely used clinical assessment standard, its integration of information regarding ulcer depth, infection, ischemia, and osteomyelitis is partly operator-dependent and cannot fully capture the independent contributions of infection burden or ischemia severity. Future work should incorporate objective imaging or wound fluid biomarkers to improve phenotypic precision. Third, the study sample comprised hospitalised patients with relatively advanced disease; selection bias may limit generalisability to ambulatory, mild DFU. Community-based validation is therefore essential. Fourth, although our multivariate model adjusted for a wide range of demographic, metabolic, and organ-function covariates, we were unable to directly control for acute-phase inflammatory markers such as C-reactive protein (CRP) or procalcitonin (PCT). A retrospective analysis of the electronic medical records in this center revealed that since most patients presented with clinically evident severe wound infections at admission, the primary treatment goal was emergency control of the infection source (debridement or amputation) rather than confirming infection through laboratory testing. Consequently, these inflammatory marker-related examinations were not routinely performed in the majority of admitted patients. Furthermore, for those patients who underwent testing later during hospitalization, the measured CRP or PCT levels could not reflect the severity of illness at admission because they had already received surgical treatment or antibiotic therapy; therefore, these markers were not included in our study. In addition, white blood cell count (another potential confounder) was highly correlated with the neutrophil percentage component of the composite indicator of interest in this study and was consequently excluded. We acknowledge that residual confounding from concurrent infection or systemic inflammatory states unrelated to DFU severity cannot be completely ruled out. Nevertheless, the Wagner grading system itself incorporates infection and osteomyelitis (grade 3) as well as gangrene (grades 4–5), thereby capturing a substantial portion of the infectious burden. Future prospective studies should systematically collect CRP, PCT, and a panel of inflammatory markers to disentangle the specific contribution of NPAR independent of acute infection. Finally, ethnic specificity cannot be ignored. Our cohort was derived from a single-center population of hospitalized patients with relatively advanced diabetic foot ulcers (DFU) in Southeast China, where dietary patterns, lifestyle habits, and genetic background may influence baseline NPAR levels. Consequently, our findings may not be directly generalizable to outpatients, patients with mild lesions (Wagner grades 0–1), or populations in primary care settings. Multicenter, community-based, and multi-ethnic validation is essential before clinical deployment.

## 5 Conclusion

In summary, this study provides the first robust evidence that the NPAR is independently and strongly associated with diabetic foot ulcer severity. The association follows a non-linear dose–response pattern, with a clinically relevant inflection point around 21.4. Integrating NPAR into routine assessment may help clinicians better recognize the current severity of DFU and guide individualised allocation of therapeutic resources. Future research should focus on translating this biomarker from bench to bedside to improve overall prognosis for patients with diabetic foot disease.

## Data Availability

The raw data supporting the conclusions of this article will be made available by the authors, without undue reservation.
