# Correction: A novel inflammatory-nutritional index: NPAR-correlates with the severity of type 2 diabetic foot ulcers

**DOI:** 10.3389/fnut.2026.1913669

**Published:** 2026-07-10

**Authors:** Kai Lin, Xiong Lei, Hongzhe Wang, Danhong Zhang, Ru Wang, Xingxing Zhang, Cai Lin

**Affiliations:** 1 Department of Burn and Wound Healing Center, The First Affiliated Hospital of Wenzhou Medical University, Wenzhou, China; 2 Department of Emergency, The First Affiliated Hospital of Wenzhou Medical University, Wenzhou, China; 3 Department of Endocrinology, The First Affiliated Hospital of Wenzhou Medical University, Wenzhou, China; 4 Wenzhou Medical University, The First School of Medicine, School of Information and Engineering, Wenzhou, China

**Keywords:** albumin, inflammatory-nutritional index, neutrophil, neutrophil-percentage-to-albumin ratio, type 2 diabetic foot ulcer severity, Wagner classification

Author [Xingxing Zhang] was erroneously omitted as equal corresponding author.

The original version of this article has been updated.

