# Supplementary material for: A novel inflammatory-nutritional index: NPAR-correlates with the severity of type 2 diabetic foot ulcers
Source: Front Nutr. 2026 Jun 18;13:1855891. doi: 10.3389/fnut.2026.1855891 (PMC13322934; doi:10.3389/fnut.2026.1855891)

Supplementary Material

A novel inflammatory-nutritional index - NPAR- correlates with the severity of type 2 diabetic foot ulcers

**Kai Lin^1^**^#^**, Xiong Lei^2^, Hongzhe Wang^1^, Danhong Zhang^3^, Ru Wang^4^, Xingxing Zhang^3^**^*^**, Cai Lin^1^**^*^

*** Correspondence:**Cai Lin, PhD.
[lincai@wmu.edu.cn](mailto:lincai@wmu.edu.cn)

**Supplementary Fig. 1 Dose response relationship between NPAR and DFU severity.**

Restricted cubic spline analysis based on ordered logistic regression


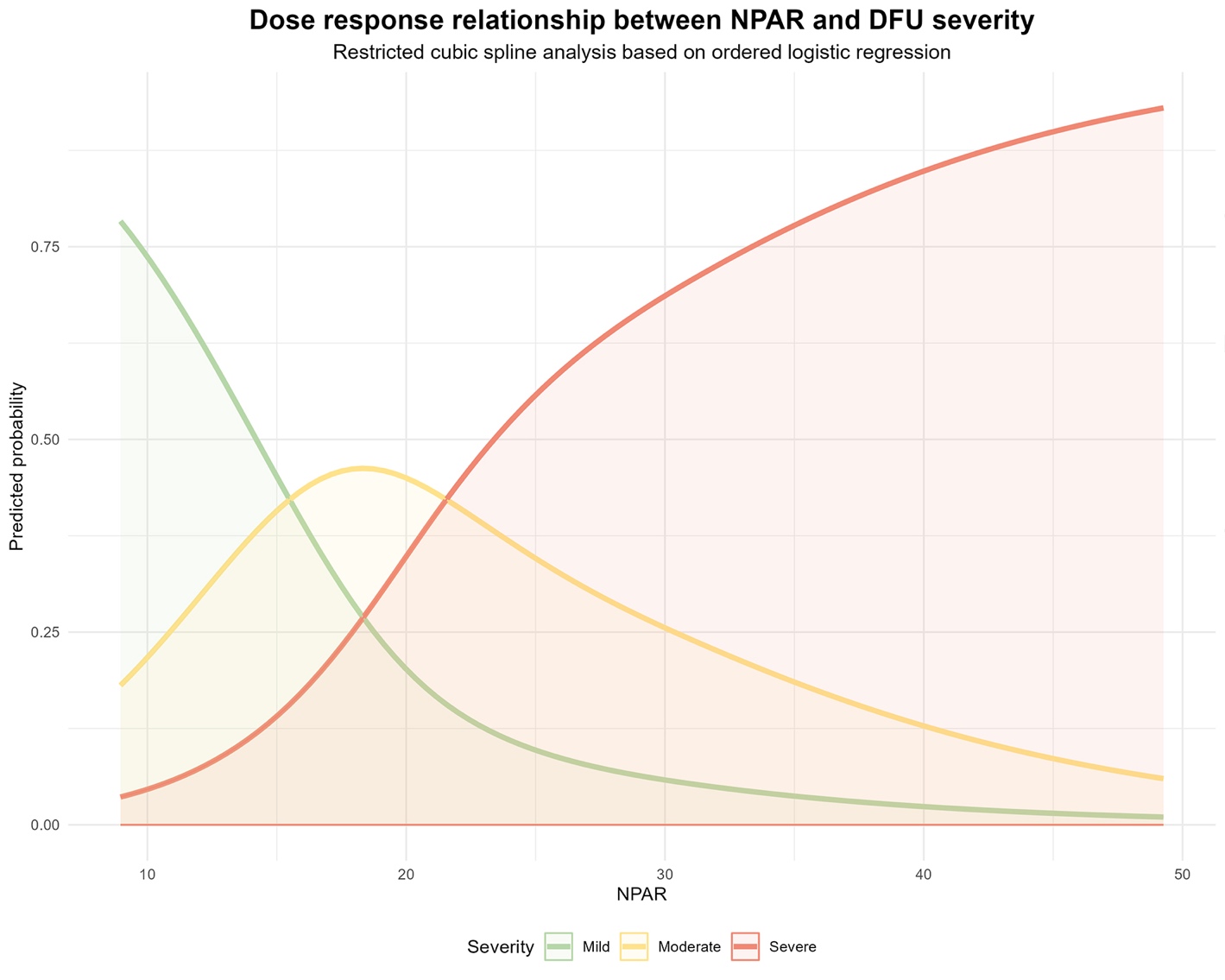


**Supplementary Fig. 2 Trend analysis of NPAR quartiles and DFU severity.**

Trend test: OR=2.214(95%Cl:1.952-2.511)


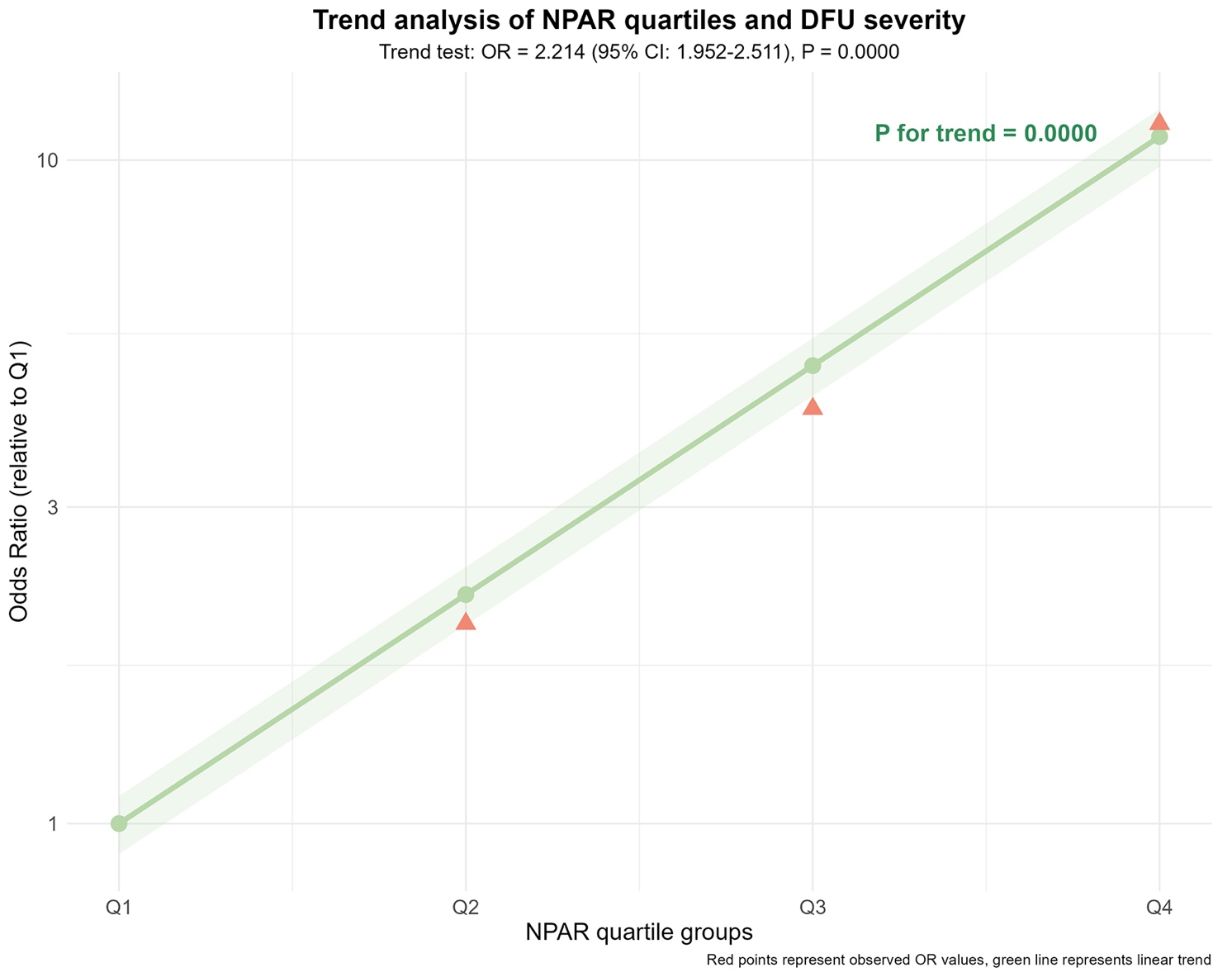

Supplement: Supplementary file 1 [file Table_1.docx]
